# SiPaGene: A new repository for instant online retrieval, sharing and meta-analyses of GeneChip^® ^expression data

**DOI:** 10.1186/1471-2164-10-98

**Published:** 2009-03-05

**Authors:** Adriane Menßen, Götz Edinger, Joachim R Grün, Ulrike Haase, Ria Baumgrass, Andreas Grützkau, Andreas Radbruch, Gerd-R Burmester, Thomas Häupl

**Affiliations:** 1 Department of Rheumatology and Clinical Immunology, Charité University Hospital, Charitéplatz 1, 10117 Berlin, Germany; 2 German Arthritis Research Center (DRFZ), Charitéplatz 1, 10117 Berlin, Germany

## Abstract

**Background:**

Microarray expression profiling is becoming a routine technology for medical research and generates enormous amounts of data. However, reanalysis of public data and comparison with own results is laborious. Although many different tools exist, there is a need for more convenience and online analysis with restriction of access and user specific sharing options. Furthermore, most of the currently existing tools do not use the whole range of statistical power provided by the MAS5.0/GCOS algorithms.

**Description:**

With a current focus on immunology, infection, inflammation, tissue regeneration and cancer we developed a database platform that can load preprocessed Affymetrix GeneChip expression data for immediate access. Group or subgroup comparisons can be calculated online, retrieved for candidate genes, transcriptional activity in various biological conditions and compared with different experiments. The system is based on Oracle 9i with algorithms in java and graphical user interfaces implemented as java servlets. Signals, detection calls, signal log ratios, change calls and corresponding p-values were calculated with MAS5.0/GCOS algorithms. MIAME information and gene annotations are provided via links to GEO and EntrezGene. Users access via https protocol their own, shared or public data. Sharing is comparison- and user-specific with different levels of rights. Arrays for group comparisons can be selected individually. Twenty-two different group comparison parameters can be applied in user-defined combinations on single or multiple group comparisons. Identified genes can be reviewed online or downloaded. Optimized selection criteria were developed and reliability was demonstrated with the "Latin Square" data set. Currently more than 1,000 arrays, 10,000 pairwise comparisons and 500 group comparisons are presented with public or restricted access by different research networks or individual users.

**Conclusion:**

SiPaGene is a repository and a high quality tool for primary analysis of GeneChips. It exploits the MAS5.0/GCOS pairwise comparison algorithm, enables restricted access and user specific sharing. It does not aim for a complete representation of all public arrays but for high quality analysis with stepwise integration of reference signatures for detailed meta-analyses. Development of additional tools like functional annotation networks based on expression information will be future steps towards a systematic biological analysis of expression profiles.

## Background

Standardization in gene expression analysis is of central importance. However, the many different concepts of bioinformatic exploitation including image processing, normalization and extraction of useful information are a matter of continuous discussion. Large repositories at the National Center for Biotechnology Information (NCBI) and European Bioinformatics Institute (EBI) archive raw data of arrays produced with different platforms [1-3]. Many different tools and strategies are available for analysis and re-analysis [4-10]. Different knowledge bases support functional interpretation [11,12]. Some tools may be specific for particular platforms; others are applicable to any platform if data are adapted to a general format.

This diversity of information and possibilities of analysis require that scientists have to familiarize themselves with laborious, time-consuming and inconvenient technical aspects, need to learn professional pre-processing, perform bioinformatic set up work and develop program specific expertise. This is time consuming and prevents many scientists from exploitation of these precious data. Furthermore, many processes of data analysis are repeatedly performed in different laboratories, individual concepts are developed and data become widely distributed but with little option to combine or share this individually generated information. Publications usually focus on particular aspects that were of interest at the time of writing the manuscript while questions on other parts of the datasets arise only thereafter.

Thus, recent approaches for example with Celsius [13], ArrayExpress [14,15], GEO profiles [16,17] or GS-LAGE [18], aim to warehouse array data in combination with various options for re-analysis. However, these databases do not offer sufficient privacy, provide in part a limited number of tools and thus are not made for primary analysis. Other database concepts focus on gene specific encyclopedic presentation of differences in signal intensity between various cell types, tissues or pathological conditions like HugeIndex [19] or relate to microarray data of a particular research field like Oncomine [20,21]. These usually do not provide the algorithms of the primary analysis used for the publication of the data. A third category of databases like BASE [22,23] used in the LCB data warehouse [24] or MARS [25] were set up to store a comprehensive list of experimental parameters and the array raw data with restricted access and to provide tools for primary analysis. However, public access in these databases is still very limited.

Finally, all databases mentioned have no options for analyses based on GCOS comparison statistics, a particular algorithm that was generated to exploit the technical specificities (multiple oligonucleotides per probe set with perfect match and mismatch sequences) of the most commonly used microarray on the market. Especially for comparison of small numbers like 5 or less arrays per group, which is a common situation, GCOS comparison statistics is a valuable tool to estimate significance of differential expression.

Being aware of the many advantages but also restrictions, we set up an Oracle 9i database called SiPaGene with the concept to store pre-analyzed array data which can be retrieved online, reanalyzed in subgroups, shared between partners and stepwise developed to a tool for functional annotations by constantly expanding information. We focused on array data generated with the GeneChip^® ^technology as a leading commercial platform and applied the standards of signal and comparison statistics implemented in the software tools MAS5.0/GCOS [26].

## Construction and content

### User and sample information

Access to the database is encrypted via https and either via anonymous public login exclusively to public data sets or via personalized and password protected login as registered users to both, public and personal data. Information about the client including name, e-mail address, login and password is stored in a table and linked to specific arrays and data. Users may access data of their own arrays or arrays generated by others who are willing to share with them.

Each array is specified by a name consisting of six different attributes to facilitate identification and recognition: 1) the donor group, 2) the type of tissue or cell that has been investigated, 3) an optional stimulus to which the tissue or cell was exposed, 4) an optional kinetic label for the stimulus, 5) the type of array used for hybridization, and 6) an incremental count that identifies each individual array of a group with the same attributes 1) to 5). These parameters describe the experiment with sufficient precision and will give future options to select specific experiments based on these attributes. The parameters are presented in an abbreviated format and stored with extended description for full text search.

Information on public arrays and experiments is provided by an array specific link to the public repository (GEO or ArrayExpress), which provides all corresponding raw data and metadata.

### Information derived from GCOS data analysis

Scanning of Affymetrix GeneChip arrays produces DAT-files that will be converted into CEL-files. The GCOS software provided by Affymetrix is specialized for the GeneChip technology with probe sets consisting of perfect- and miss-match oligonucleotides. We experienced advantages of this analysis concept especially for comparison of small sample sizes. Based on this software and its standard settings for detection and change calls, we perform signal analysis by global normalization and scaling to a target value of 150 for all array types. For each array, signal values, detection calls and detection p-values are exported from GCOS as txt-files and imported into a specific table in the SiPaGene database. This table is linked to the name of the array, probe set IDs, and information about ownership and sharing with other users.

The algorithm for pairwise array comparison in the GCOS software provides signal log ratios (SLR), change calls and change p-values. These three parameters that are calculated for each probe set, are imported into the comparison table in the SiPaGene database and are linked to the name of the experiment and the baseline array as well as to a table with ownership and sharing status for pairwise comparisons.

### Group comparisons

Group comparisons are performed between two groups of arrays and combine data from pairwise comparisons and signal information from all arrays combined to one group. Names of these group comparisons are deduced from the 6 attributes that define the names of the arrays involved. These names are stored in a separate description table and are linked to the tables that contain the array names, the pairwise comparisons and the ownership and sharing information for group comparisons and pairwise comparisons. Thus, the right for access to the complex ownership information for a group comparison is immediately linked to the name of a group comparison, which facilitates sharing from the perspective of the user. To start the first group comparison for two groups of arrays, access is restricted exclusively to the owner by the ownership status of all pairwise comparisons needed for a group comparison.

### Information generated by group comparisons

The different parameters derived from signal calculation by the GCOS software were used to calculate mean, median and standard deviation of signal values as well as the percentage of "present" calls for the groups of experiment arrays and baseline arrays separately. We included "marginal" calls into the number of "present" calls. Calculating the mean of the Signal Log Ratio (SLR) values, the conversion of this value into the fold change, and the percentage of „increased“ and „decreased“ calls summarizes pairwise comparison information of all possible comparisons. Furthermore, the percentage of "no change" calls is calculated between all experiment and baseline arrays, between all arrays of the experiment group and between all arrays of the baseline group. Finally, different Welch t-tests are performed: i) with the log transformed signal values between experiment and baseline group, ii) with SLR values by comparing the SLR of the experiment versus baseline group with the SLR of the comparisons either within the experiment or the baseline group alone or with the SLR of both group-internal comparisons together. All these data are stored in the table for group comparisons, which is linked to the name of the group comparison.

### Ownership and data sharing

To define ownership and data sharing between users, array-specific and comparison-specific ownership and sharing information is necessary. This is in accordance with our concept that group comparisons between sets of arrays are the core information stored in and retrieved from the SiPaGene database. The rights to access data are classified in 4 groups: 1) a user is owner of the array and the pairwise comparison; 2) sharing allows another user, who is defined by the owner, to retrieve candidate genes from group comparisons (read a group comparison); 3) the previous sharing level is extended to the right that new group comparisons with sub-selections of arrays from a shared group comparison can be regrouped and a new comparison can be initiated (write a group comparison); 4) the user defined by the owner has all rights of the owner including the rights to give other defined users access to the shared group comparison. All other users that are not explicitly the sharing partners of the owner have no access to the data. The owner can revoke sharing rights at any time and can never be excluded from access to his own arrays and comparisons.

### Annotation of gene and array information to each probe set

All signal and comparison information is linked to its particular probe set. Each probe set is linked to gene annotation and ontology data as provided by Affymetrix. Additional links are connecting to Entrez Gene entries of the corresponding gene.

**Figure 1:**
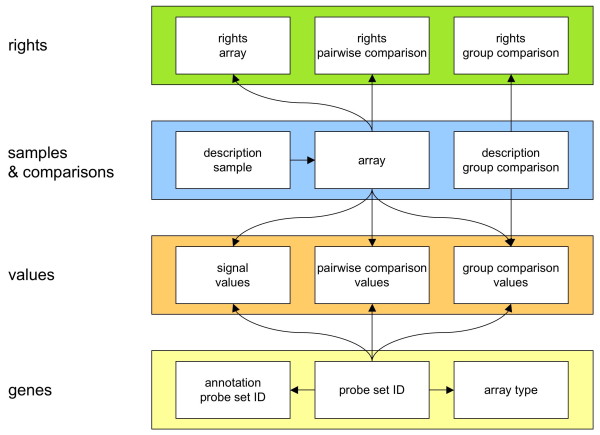
**Overview of the information stored in the SiPaGene database: The four different categories of information about rights, samples and comparisons, values and genes are linked as relational network**. Arrows indicate that individual subcategories are connected via primary key identifiers. This demonstrates that arrays and probe set IDs are the central anchors for the huge lists of signal and comparison values.

All together, arrays and probe set IDs are the central anchors for all signal and comparison values as well as information about genes, comparisons and rights of access (figure 1).

## Utility

### General aspects, access and administration

**Figure 2:**
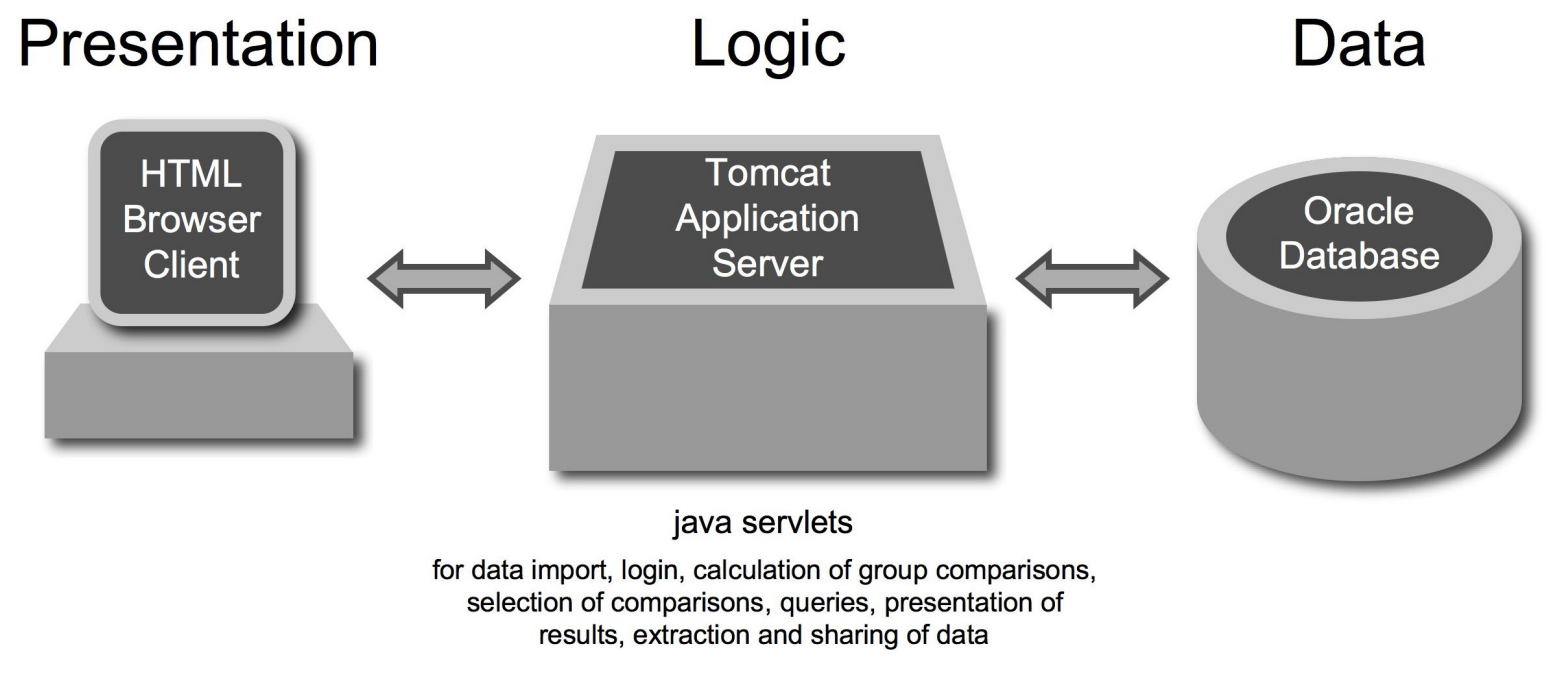
**Three-tier model with secure login and exchange of information between the client and the SiPaGene database**. Java servlets provide graphical user interfaces to perform comparisons and queries or to organize sharing information.

A three-tier architecture was applied to interact with the SiPaGene database (figure 2). Special diligence was applied to respect privacy of data and to offer several options to share with other clients. With an Internet browser and a secure http connection , the client contacts the Tomcat server to create his own account, to login and to work via graphical user interfaces (GUI) programmed as java servlets. The different GUIs allow access for a registered or an anonymous (public) user. For personal accounts, after registration, a random password will be sent by e-mail that can be changed by the client. Three incorrect attempts to login will block the account and a new random password with activation link will be sent to the client's e-mail account. Passwords are stored MD5-encrypted in the database. User specific sharing is only possible with personal accounts. Access enables to perform new group comparisons, to search and select from existing group comparisons, to select different types of default queries, to define query parameters individually and to explore candidate genes with different options. Administration is performed directly with the oracle server. This includes generating the GCOS data analysis of individual arrays and pairwise comparisons, importing the data with a batch processing java program, managing the user accounts, and administrating the general aspects of oracle 9i (figure 2).

### Perform a group comparison

To analyse gene expression data, group comparisons have to be performed. These are initiated by selecting in three steps the experiment group, the baseline group and the individual arrays for each group. Thereafter, a java program calculates the values of the 17 different parameters for the group comparison as outlined in the description of the structure. As soon as the calculation process is completed, the name of the group comparison will appear in the selection menu for group comparison queries.

### Find group comparisons of interest

A growing number of public data sets are available and can be searched and selected for analysis in the „find“ area. Queries can be performed globally or in each of the six annotation categories with free text search fields or by selecting from the list of abbreviated annotations. Furthermore, array- or experiment-IDs from the GEO public repository can be retrieved. All group comparisons associated with such query parameters will be shown and can be tagged to narrow down the number of comparisons of interest. Depending on the selected option, all accessible, all found, or only the tagged comparisons will be shown in the drop-down menus of the subsequent pages for choosing group comparisons for analysis.

### Perform a query

There are two options to retrieve candidate genes, using either the single group comparison or the multiple group comparisons. The query form for single group comparisons displays all different query parameters and allows to combine these by the Boolean operator AND for all parameters in the same column as well as with OR or NOT in different columns (up to 5). The different parameter fields offer to select for candidate genes by individual GeneChip probe set IDs, gene title, gene ontology annotations, cut-off levels for signal intensities and the frequency of significant detection for each group. Furthermore, thresholds for parameters of comparative statistics may be applied. These include the mean value of all pairwise SLR results from *n *× *m *comparisons with *n *and *m *representing the number of arrays in the experiment and the baseline group, respectively. The fold change (*FC*) parameter is deduced from the SLR value by the standard conversion *FC *= 2^*SLR *^for SLR = 0 and *FC *= 2^-*SLR *^for SLR < 0 and thus represents an alternative parameter for the same type of information. Another set of parameters combines information about the frequency of differential expression between both groups. These consist of the percentage of increased, decreased, or not changed expression of all *n *× *m *comparisons as well as not changed expression between arrays of only the experiment or only the baseline group. These parameters help to identify candidate genes more or less consistent in differential expression. Finally, Welch t-tests can be retrieved, one based on signal information, three based on SLR information as outlined above. The advantage of the SLR based t-tests is that more measure points are included. Thus, for small sample groups statistical interpretation is stabilized and strict Bonferroni correction for these tests is effective to minimize false discovery. These tests are helpful for the refinement of the selection process especially in comparisons with minor differences. Based on practical experience [27], default filter strategies were developed and programmed for convenient selection of increased and decreased candidate probe sets.

The multiple group comparison page allows selecting and comparing candidate genes from different group comparisons using all parameters described above. Each parameter of a group comparison can be combined with another parameter of the same or another group comparison with AND, OR or NOT. Up to 20 different combinations are currently possible.

### Candidate genes

The probe sets identified by either of the two types of queries are displayed in a table consisting of the different query parameters except from gene ontology but including a link to Entrez Gene information. This table can be downloaded as txt- or Excel-file. Furthermore, the signals of each array and candidate probe set can be downloaded as separate lists in txt- or Excel-format for additional analyses like clustering. Each probe set ID is linked to a detailed outline of all parameters and signals of each individual array of the comparison.

### Management of ownership, data sharing and comparisons

Each array, pairwise and group comparison is linked to ownership and sharing information. The client who owns the data can define and control all sharing conditions. The GUI for "Rights" requires to identify the collaboration partner and subsequently to define the status of sharing for each group comparison owned by the client. This will transfer also the corresponding rights to access pairwise comparisons if the collaboration partner is allowed to perform subgroup analyses of a group comparison. Thus access is exclusively restricted to the particular set of data shared with the selected partner.

If comparisons are no more of interest and will not be used in the future, clients are encouraged to delete this information to increase efficiency of data storage and retrieval. The page to manage comparisons is accessible via the site "Comparison" and displays only comparisons initiated by the client himself.

**Figure 3:**
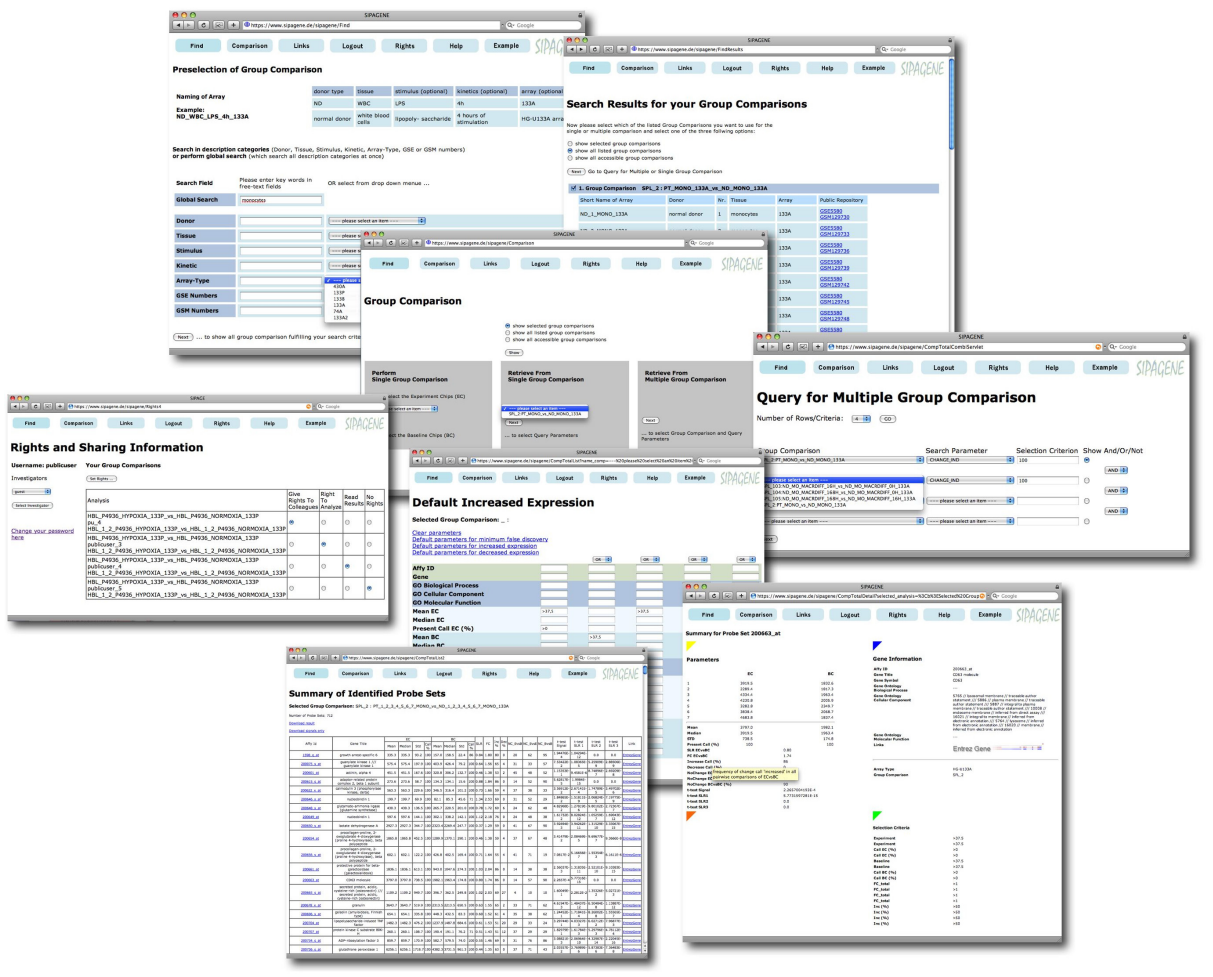
**An overview of the most frequently used graphical user interfaces: The find GUI enables to retrieve and to select comparisons of interest for further analysis and provides access to experiment information**. The group comparison GUI allows entering the areas to perform or to query group comparisons. Query parameters may be selected individually or by default. Output of candidate genes is summarized in a list of identified probe sets, may be downloaded or viewed in detail for individual probe sets. Sharing of group comparisons is managed via the GUI for rights and sharing information for each named user individually.

In figure 3 the most important graphical user interfaces are summarized.

### Case study: The Affymetrix Latin Square spike-in data set

The Latin Square experiment provided by Affymetrix [28] is a relevant example to test the database. It consists of no more than triplicates and therefore reflects a frequent situation of current profiling experiments. With specific mRNAs of known concentration spiked into the different experiment samples in a Latin Square format, it enables to determine the performance of the query options of SiPaGene. In detail, 14 groups of triplicate experiments (≙42 arrays) with transcriptomes of the HeLa cell line (ATCC CCL-13) are spiked with various concentrations of 14 × 3 different mRNAs (table 1). These 42 mRNAs are not expressed in the HeLa transcriptome and represent artificial sequences, or eukaryotic controls, which are detectable by complementary probe sets on the HG-U133Atag array. They interfere with 22 additional probe sets of the HG-U133Atag array as described previously [29] and consist of at least 9 of the 11 oligonucleotides complementary to any of the spikes. An additional probe set exists (205397_x_at), which was already described by Affymetrix but has reduced binding specificity with only 5 out of 11 oligonucleotides complementary to the spike for probe set 205398_s_at. Within all 22300 transcripts, the fraction of true positives (TP) consists of 65 probe sets (including 205397_x_at).

**Table 1:** Latin Square experiment

**Group ID**	**Gene ID of spiked sequence**	**EXP 1**	**EXP 2**	**EXP 3**	**EXP 4**	**EXP 5**	**EXP 6**	**EXP 7**	**EXP 8**	**EXP 9**	**EXP 10**	**EXP 11**	**EXP 12**	**EXP 13**	**EXP 14**
1	203508_at 204563_at 204513_s_at	0	0.125	0.25	0.5	1	2	4	8	16	32	64	128	256	512
2	204205_at 204959_at 207655_s_at	0.125	0.25	0.5	1	2	4	8	16	32	64	128	256	512	0
3	204836_at 205291_at 209795_at	0.25	0.5	1	2	4	8	16	32	64	128	256	512	0	0.125
4	207777_s_at 204912_at 205569_at	0.5	1	2	4	8	16	32	64	128	256	512	0	0.125	0.25
5	207160_at 205692_s_at 212827_at	1	2	4	8	16	32	64	128	256	512	0	0.125	0.25	0.5
6	209606_at 205267_at 204417_at	2	4	8	16	32	64	128	256	512	0	0.125	0.25	0.5	1
7	205398_s_at 209734_at 209354_at	4	8	16	32	64	128	256	512	0	0.125	0.25	0.5	1	2
8	206060_s_at 205790_at 200665_s_at	8	16	32	64	128	256	512	0	0.125	0.25	0.5	1	2	4
9	207641_at 207540_s_at 204430_s_at	16	32	64	128	256	512	0	0.125	0.25	0.5	1	2	4	8
10	203471_s_at 204951_at 207968_s_at	32	64	128	256	512	0	0.125	0.25	0.5	1	2	4	8	16
11	AFFX-r2-TagA_at AFFX-r2-TagB_at AFFX-r2-TagC_at	64	128	256	512	0	0.125	0.25	0.5	1	2	4	8	16	32
12	AFFX-r2-TagD_at AFFX-r2-TagE_at AFFX-r2-TagF_at	128	256	512	0	0.125	0.25	0.5	1	2	4	8	16	32	64
13	AFFX-r2-TagG_at AFFX-r2-TagH_at AFFX-DapX-3_at	256	512	0	0.125	0.25	0.5	1	2	4	8	16	32	64	128
14	AFFX-LysX-3_at AFFX-PheX-3_at AFFX-ThrX-3_at	512	0	0.125	0.25	0.5	1	2	4	8	16	32	64	128	256

**Figure 4:**
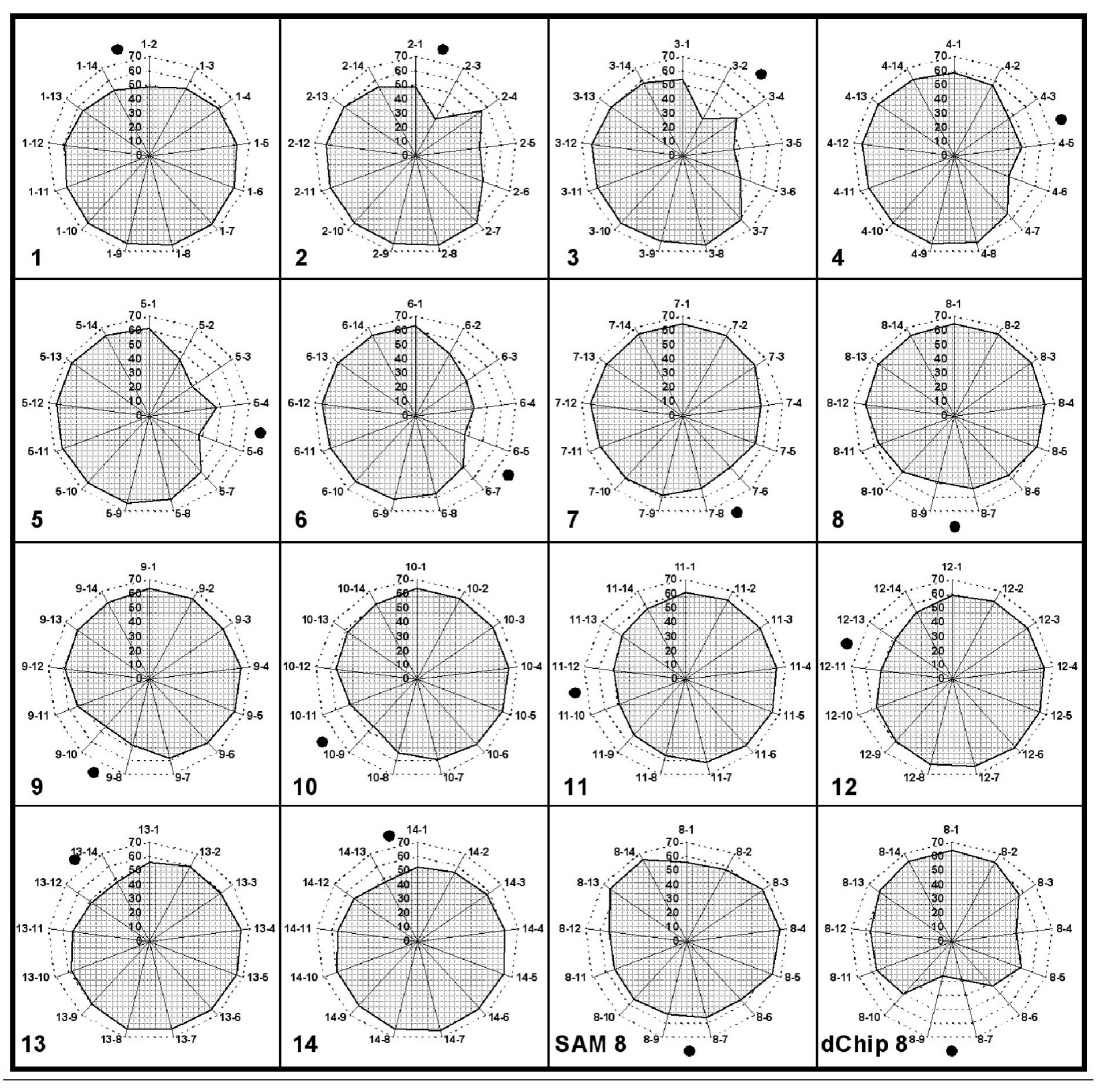
**The spider charts 1 to 14 (referring to experiments 1–14) present the number of true positive probe sets that were identified by the SiPaGene optimized filter strategy**. The number of the chart indicates the Latin Square experiment that was compared to all other 13 experiments. The comparisons are displayed in the sequence of the Latin Square spiking groups 1 to 14 in clockwise order. For example, in spider chart 1, the Latin Square experiment 1 was compared to all others. The label "1–2" indicates the comparison of experiments 1 with experiment 2. Here close to 50 of the 65 true positive probe sets were identified. The charts "SAM 8" and "dChip 8" present the recall of true positives comparing the Latin Square experiment 8 with all other experiments and applying the software tools SAM and dChip, respectively. The black dot in each chart is located between the comparisons of the Latin Square group as indicated by the chart number with the experiments next to it in the Latin Square table. These are the comparisons with only one titer step of difference in the concentration of the spiked sequences. Therefore, the dot indicates those comparisons that are expected to present with the lowest recall rates. On the other hand, opposite to the dot, the highest recall rates can be expected and were indeed identified with 60 or more true positives.

**Figure 5:**
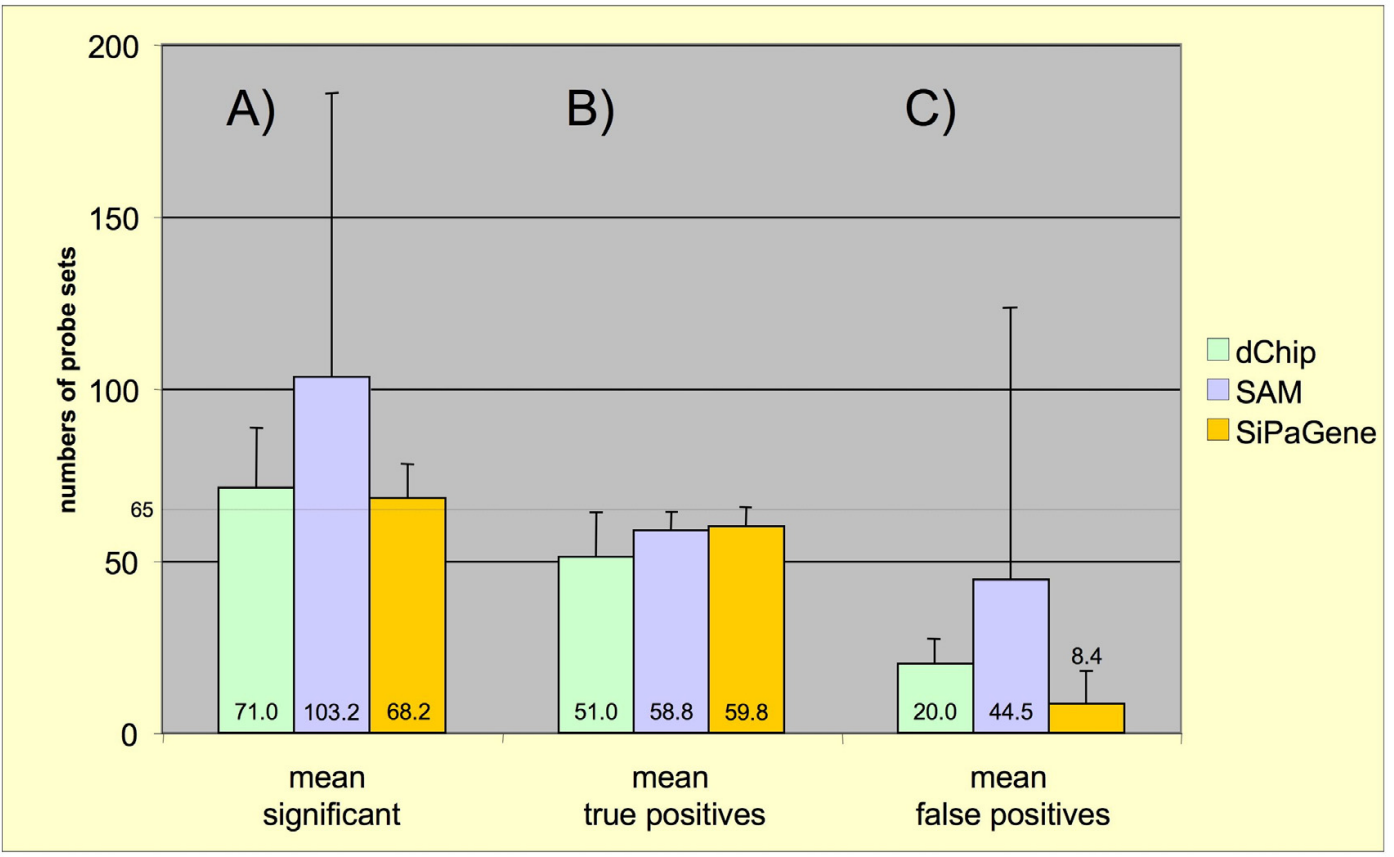
**Comparisons of recall rates between dChip, SAM, and SiPaGene**. Experiment 8 was compared to all other experiments with all three tools. A) The mean and standard deviation (SD) of all probe sets identified by the different tools is displayed. Part B) presents the mean and SD of correctly recalled probe sets and part C) the mean and SD of false positive probe sets. The optimized filter strategy implemented in SiPaGene identified the number of probe sets with the highest recall and the lowest error rate. The relatively poor outcome of SAM results from a singular high false positive recall between experiment 8 and 4 (n = 326) indicating that this algorithm may fail in few exceptions.

Concentrations (pmol) of spiked sequences for all 14 experiments. Details and sequences are presented on the Affymetrix web site [28]. Seven of the 42 spikes affect 23 additional probe sets of the HG-U133Atag array, thus increasing the number of affected probe sets on the HG-U133Atag array to 65. The spike for probe set 205398_s_at in group 7 consists of sequence elements identical to 5 of the 11 oligonucleotides of probe set 205397_x_at. Thus, comparing experiment 8 with all other experiments has the best chance to detect interference with probe set 205397_x_at (Figure 5). The sequences spiked in group 14 interfere with the here shown 3 and 15 additional probe sets. This spike group has the lowest concentrations in experiments two to six (0 to 1.0 pmol), resulting in the lowest recall rates in these comparisons (cf. figure 4, chart number 2 – 6). In these comparisons not only 3 but 18 true positives have very low concentrations and are difficult to detect.

To compare all 14 different experimental groups with each other, 91 (= 14 × 13/2) group comparisons were necessary. Each group comparison consisted of 9 (= 3 × 3) pairwise comparisons between two groups and the three comparisons within each group, which are applied in both directions. Thus 42 arrays were analyzed with GCOS to generate the signals, detection calls, detection p-values and 861 different pairwise comparisons. All data were imported into SiPaGene and then grouped to calculate the values of the query parameters for the 91 comparisons.

### Case study: Queries and identification of transcripts with SiPaGene

An optimized filter strategy was developed to exclude false positives based on high statistical variation and to include the true positives based on stable differential expression. This query results from independent investigations with own array experiments (e.g. [27]) and consists of a cut-off for low signals, a threshold for statistical significance of detection, a threshold for homogeneity of change and a Bonferroni corrected measure of statistical significance (e.g. for the Latin Square dataset a t-test p value below 1.49E-7 was used). It is implemented in the database with three different default queries that were merged to the union of all three as the optimized selection.

This query correctly identified most of the 65 true positive probe sets as shown in figures 4 and 5. The transcript AFFX-r2-TagE_at was identified most frequently (90 of 91 comparisons; not found only in Exp7 vs. Exp6, i.e. 0.5 vs. 0.25 pmol; cf. table 1). The least frequently identified probe set was 205397_x_at (38 times), which has only 5 of 11 oligonucleotides complementary to the spike for the target probe set 205398_s_at. All other true positives were identified in at least 69 of the 91 comparisons. The four false positives with the highest frequency of identification in the 91 comparisons were the transcripts 203173_s_at (49 calls), 204890_s_at (46 calls), 204891_s_at (45 calls), and 213060_s_at (25 calls). All other false positives were found in 18 comparisons or less and the majority (73 transcripts) only once. Even the minimum concentration of 0.125 pmol compared to no spike was identified, but only for two transcripts (AFFX-r2-TagH_at and AFFX-r2-TagE_at) which were in independent spike groups.

### Case study: Comparing SiPaGene with SAM and dChip

We compared our filter strategy with the DNA-chip analyzer dChip (version June, 27^th ^2005) [5] and the significance analysis of microarrays (SAM, version 1.15) [6]. dChip provides its own normalization of GeneChip array data and was used with correction for mismatch oligonucleotide hybridization. SAM was applied on RMA-Express data [4,30,31]. The delta value of the SAM tool was set to the thresholds were the number of false positive genes is calculated as = 1 and the median false discovery rate is minimal.

Experiment 8 was selected for comparison, because the spike for 205398_s_at was maximum (512 pmol, Table 1) and, therefore, had the best chance to interfere with probe set 205397_x_at to identify all 65 true positives. Experiment 8 was compared with all other 13 experiments and the total number of identified probe sets as well as true and false positives was counted. Figure 5 presents the mean and standard deviation of the results for all three tools and 13 group comparisons, each. SAM had a high recall ratio for true positives but failed in a few comparisons to eliminate false positives as reflected by the high standard deviation (worst in the comparison of Exp 8 versus Exp 4 with more than 300 hits). dChip revealed slightly less true positives and presented a small but constant selection of false positives. Compared to SAM and dChip, the optimized query with SiPaGene produced the best selection with most of the true positives and the least of the false positives. In detail, at least 60 true positives were identified with dChip only in four, with SAM in five and with SiPaGene in 8 of the 13 comparisons. In all other analyses (1 – 7 and 9 – 14) not directly compared with SAM or dChip, the optimized query implemented in the SiPaGene database identified this high recall rate of 60 or more true positives in at least 7 of the 13 comparisons performed in each experiment group (figure 4). In summary, although differences are not extreme between SAM, dChip and SiPaGene, the study demonstrates that a complex query can improve the rate of correctly identified genes and that this query can be standardized independently of another experiment.

## Discussion

SiPaGene provides rapid GeneChip analysis based on MAS5.0/GCOS statistics with a standardized workflow to generate various statistical parameters for optimized but also flexible selection conditions. An important feature is the management of access with administrative tools to define for each comparison absolute privacy, different levels of user specific sharing or full public access. Thus, the SiPaGene database combines the functions of a repository for gene expression data with tools for flexible and optimized primary as well as meta-analysis with single and multiple group comparisons. Increasing numbers of public and private data sets along with the sharing options enable validation, improve interpretation and encourage controlled exchange of array data.

Other algorithms like RMA have replaced MAS5.0/GCOS pairwise comparison statistics. Previous reports demonstrated that signal normalization by these newer algorithms improve results of comparative analysis [29,32]. However, these tools have been compared with MAS5.0/GCOS on the basis of probe set signal calculation. Here we could show exemplarily with the data of the Latin Square spiking experiment that a consequent application of the MAS5.0/GCOS pairwise comparison statistics provides more robust results than analyses with dChip or with RMA and SAM.

Many GeneChip array experiments in GEO or ArrayExpress were performed with a limited number of hybridizations (usually less than 5 arrays per group). Two principle factors influence statistical power in these experiments: biological and technical variability. While testing of biological variability cannot be improved except by increasing the number of array hybridizations, technical variability can be assessed using the GeneChip information from individual oligonucleotides of each probe set not only for signal calculation but also for statistical testing of differential expression between two arrays. This extended information is provided by the change call statistics in MAS5.0/GCOS for pairwise comparisons and is summarized in the derivative parameters calculated in SiPaGene for each group comparison. The exponentially increasing number of pairwise comparisons with increasing numbers of arrays per group is certainly a disadvantage and a limit of this approach. For example, comparing two groups of 10 arrays each will require calculation of 190 pairwise comparisons, two groups of 50 arrays each already 4950 pairwise comparisons. However, this is a rare problem and calculating and importing up to 5000 pairwise comparisons for such a particular experiment is not out of reach. Furthermore, these larger groups are often clustered in subgroups, which can be conveniently further investigated by calculating new group comparisons of these subgroups because all relevant pairwise comparisons are already imported in SiPaGene for the full group comparison.

Concerning performance of queries, the first selection step from the table storing all probe set information from a group comparison is based on the index pointing to the name of the group comparison. All other conditions are without index and subsequently retrieved out of the number of all probe sets per comparison. Thus, performance of the database will be challenged when the number of group comparisons is substantially higher than the number of probe sets per array.

The main object of SiPaGene is immediate access to the results of MAS5.0 and GCOS statistics. Raw data and experimental metadata are maintained in other excellent platforms and are therefore linked for all public data mainly from GEO. This concept was favored to harmonize information and to avoid work for already existing and constantly curated information.

Considering that for the majority of experiments only a small subset of transcripts is changing, the global normalization method implemented in the MAS5.0/GCOS software was applied to scale all arrays to a constant overall intensity. This enables to constantly expand the number of arrays without renormalization. Currently favored algorithms like RMA require renormalization of the whole set of arrays with each additional array to allow comparison between all arrays. Therefore, it has been suggested to normalize each array to a set of reference arrays [33]. This seems to overcome the initial limitation when we were starting to setup SiPaGene and thus may offer to integrate other algorithms like RMA in the future.

SiPaGene was set up as a database that combines both, high quality of retrieval options not only for specialists in bioinformatics and storage of the growing number of microarray experiments for meta-analyses. It was developed for the Affymetrix GeneChip platform technology and allows rapid and automated calculation for experiments with many different group or subgroup comparisons. The quality of optimized queries was tested using the Latin Square experiment provided by Affymetrix. This data set has frequently been used to optimize bioinformatic tools for microarray data analysis [32]. We could demonstrate that the MAS5.0/GCOS primary signal and pairwise comparison analysis provide a solid basis to identify the relevant candidate genes. Sensitivity was only decreasing when spike concentrations were very low (cf. charts 2 – 6 in figure 4) but this was also observed with dChip or with RMA and SAM. Especially, all comparisons of the experiments two to six, which affected the highest number of interfering probe sets (n = 15 out of 23, spike group 14) with the lowest spike concentrations for group 14 (0 – 1.0 pmol), revealed the lowest recall rates for true positives. Nevertheless, the optimized filter strategy of SiPaGene could outperform standard tools like SAM [6] and dChip [34]. It revealed an excellent recall rate for true positives and the lowest rate for false positives even for the small replicate number of three arrays per group. This demonstrates that MAS5.0/GCOS algorithms with normalization of each array separately and statistics based on many different oligonucleotide probes per probe set are highly effective to identify the relevant differences.

Another important option in the SiPaGene database is the possibility to restrict access and to enable user- and comparison-specific sharing of data. Many expression studies have been published without submission of the related raw data to any of the public repositories. This indicates that scientists are very conservative in terms of sharing their data freely. One important reason for this seems to be the limitation to interpret the biological processes despite holding a genome-wide transcription profile in hands. There is hope that appropriate tools to elucidate the function behind these data will improve constantly and give much better insight within the next few years. Based on own experience, functional interpretation improves with the number of comparisons performed with different if possible defined reference signatures and therefore is a cornerstone for future array analysis [35]. Such signatures depend on high quality experiments and will be the least ones to be shared publicly. Therefore, tools are needed that encourage collaborative exchange and thereby enable the development of new and better tools for interpretation of expression data.

Based on the tools for detailed group analysis of individual GeneChip experiments, options to analyze multiple group comparisons were integrated. These are indispensable to perform meta-analyses. With a growing set of reference signatures, it will be possible to define the degree of specificity of individual genes for a defined biological function and to develop signature based functional annotation tools. These are important and complementary to existing annotation and interpretation software based on literature information about individual genes, gene interactions and biological functions [12,36,37]. Generating annotations based on such meta analyses, this information can be immediately sourced to experimental data while literature based annotations depend on the quality of assignment and are often longsome and difficult in tracing back.

Next improvements, which are currently in preparation, will include an expanded functionality, such as tools for visualization (clustering), upload and administration of gene lists for comparative retrieval with predefined candidate genes, selection and storage of marker genes for quantification of cell-type and stimulus-specific signatures and to enable users to define expression-based annotations.

## Conclusion

Currently, about 100 registered users have access to about 1,000 arrays (≈500 for public access), 10,000 pairwise comparisons and more than 500 group comparisons. In three large research networks with national and international collaborations, sharing and ownership restrictions proved their value to communicate data between defined partners and to perform individual and user-specific queries immediately after hybridization. The current version does not aim for full implementation of any public GeneChip data set like large warehousing databases do. The intension is to provide an easily accessible high quality framework for analysis and sharing and to enable stepwise integration of relevant reference data according to the needs of the users. The current set of open access data is derived from public repositories, focuses predominantly on immunology, inflammation, infectious diseases, tissue regeneration and cancer, and includes studies performed with tissues, blood or isolated cells. The various biological conditions represented by these experiments can be stepwise integrated in meta-analyses, can be exploited for the generation of functional annotation networks and may provide a basis towards a systems biology approach for the interpretation of gene expression profiles.

## Availability and requirements

Access to the Latin Square data and public data sets is open and provided via . Academic institutions may request the software package from the authors for own non-commercial use. Non-academic commercial use is restricted. For licensing please contact the Charité, Technology Transfer, Charitéplatz 1, 10117 Berlin.

## Authors' contributions

AM developed the basic schema of SiPaGene, the corresponding 3-tier-system, programmed and administrated together with GE and UH the different parts of the database and drafted main parts of the manuscript. JG performed the case study and together with AG and RB he contributed with definitions of search parameters, optimized filter strategies, Bonferroni corrected SLR Welch t tests and helped to draft the manuscript. AR and GRB helped to design data sharing for research networks and to draft the manuscript. TH conceived of the project, participated in its design and coordination and helped to draft the manuscript. All authors read and approved the final manuscript.

## Funding of authors

AM was funded by the NGFN and GenoStem, GE by the NGFN, and JG, AG and UH by the NGFN and AutoCure. AR and RB are employed by the DRFZ, GRB and TH by the Charité.
